# Does induction or augmentation of labor increase the risk of postpartum hemorrhage in pregnant women with anemia? A multicenter prospective cohort study in India

**DOI:** 10.1002/ijgo.16008

**Published:** 2024-11-08

**Authors:** Tuck Seng Cheng, Farzana Zahir, Carolin Solomi, Ashok Verma, Sereesha Rao, Saswati Sanyal Choudhury, Gitanjali Deka, Pranabika Mahanta, Swapna Kakoty, Robin Medhi, Shakuntala Chhabra, Anjali Rani, Amrit Bora, Indrani Roy, Bina Minz, Omesh Kumar Bharti, Rupanjali Deka, Charles Opondo, David Churchill, Marian Knight, Jennifer J. Kurinczuk, Manisha Nair

**Affiliations:** ^1^ National Perinatal Epidemiology Unit, Nuffield Department of Population Health Oxford University Oxford UK; ^2^ Department of Obstetrics and Gynaecology Assam Medical College Dibrugarh Assam India; ^3^ Department of Obstetrics and Gynaecology Makunda Christian Leprosy and General Hospital Karimganj Assam India; ^4^ Department of Obstetrics and Gynaecology Dr Rajendra Prasad Government Medical College Tanda Himachal Pradesh India; ^5^ Department of Obstetrics and Gynaecology Silchar Medical College and Hospital Silchar Assam India; ^6^ Department of Obstetrics and Gynaecology Gauhati Medical College and Hospital Guwahati Assam India; ^7^ Department of Obstetrics and Gynaecology Tezpur Medical College Tezpur Assam India; ^8^ Department of Obstetrics and Gynaecology Jorhat Medical College and Hospital Jorhat Assam India; ^9^ Department of Obstetrics and Gynaecology Fakhruddin Ali Ahmed Medical College and Hospital Barpeta Assam India; ^10^ Department of Obstetrics and Gynaecology Mahatma Gandhi Institute of Medical Sciences Sevagram Maharashtra India; ^11^ Department of Obstetrics and Gynaecology Banaras Hindu University Institute of Medical Sciences Varanasi Uttar Pradesh India; ^12^ Department of Obstetrics and Gynaecology Sonapur District Hospital Guwahati Assam India; ^13^ Department of Obstetrics and Gynaecology Nazareth Hospital Shillong Meghalaya India; ^14^ Department of Obstetrics and Gynaecology Sewa Bhawan Hospital Society Basna Chattisgarh India; ^15^ Department of Health & Family Welfare, State Institute of Health and Family Welfare Government of Himachal Pradesh Shimla Himachal Pradesh India; ^16^ MaatHRI Project Srimanta Sankaradeva University of Health Sciences Guwahati Assam India; ^17^ Department of Medical Statistics London School of Hygiene & Tropical Medicine London UK; ^18^ Department of Obstetrics and Gynaecology The Royal Wolverhampton NHS Trust Wolverhampton UK; ^19^ Research Institute for Healthcare Science University of Wolverhampton Wolverhampton UK

**Keywords:** anemia, clinical indication, labor induction/augmentation, postpartum hemorrhage

## Abstract

**Objective:**

To investigate whether induction/augmentation of labor in pregnant women with anemia increases the risk of postpartum hemorrhage (PPH) and whether this risk varied by indications for labor induction/augmentation and by anemia severity in pregnancy.

**Methods:**

In a prospective cohort study of 9420 pregnant women from 13 hospitals across India, we measured hemoglobin concentrations at recruitment (≥28 weeks of gestation) and blood loss after childbirth during follow‐up and collected clinical information about PPH. Clinical obstetric and childbirth information at both visits were extracted from medical records. Anemia severity in the third trimester was categorized using hemoglobin concentrations (no/mild anemia: hemoglobin ≥10 g/dL; moderate: hemoglobin 7 to 9.9 g/dL; severe: hemoglobin <7 g/dL), while PPH was defined based on blood loss volume (vaginal births: ≥500 mL or cesarean sections: ≥1000 mL) and clinical diagnosis. Indications for labor induction/augmentation were classified as clinically indicated and elective as per guidelines. We performed multivariable modified Poisson regression analyses to investigate the associations of anemia severity and indications for labor induction/augmentation, including their interaction, with PPH, adjusted for potential confounders.

**Results:**

PPH was associated with anemia but not with indications for labor induction/augmentation. However, there was a significant interaction between the two factors in relation to PPH (*P* = 0.003). Among pregnant women with severe anemia, a higher risk of PPH was associated with elective (adjusted risk ratio, 3.44 [95% confidence interval, 1.29–9.18]) but not with clinically indicated (adjusted risk ratio, 1.22 [95% confidence interval, 0.42–3.55]) labor induction/augmentation. No associations were observed among pregnant women with no/mild and moderate anemia.

**Conclusion:**

The risk of PPH is higher in women who have moderate–severe anemia in late pregnancy. Induction/augmentation of labor is generally safe for women with anemia, but it can increase the risk of PPH in women with severe anemia if performed electively.

## Synopsis

Anemia in late pregnancy increases the risk of postpartum hemorrhage, and the risk is substantially higher in women with severe anemia who are induced/augmented without a clinical indication.

## 1 INTRODUCTION

Induction and augmentation of labor are widely used labor interventions around the world, including in low‐ and middle‐income countries.^1^, ^2^, ^3^ These procedures are usually performed when the benefits to maternal and/or fetal health by hastening childbirth outweigh the risks associated with a continued pregnancy or prolonged labor. Decisions about labor induction/augmentation are generally guided by specific clinical indications and contraindications as outlined in international and national guidelines.^4^, ^5^, ^6^ Various methods, including mechanical approaches, pharmacological agents, or a combination of both are used with the aim of initiating and accelerating uterine contractions.^5^ However, these may elevate the risk of inappropriate stimulation of the uterus during or after childbirth, leading to severe bleeding, known as postpartum hemorrhage (PPH), which is a leading direct cause of maternal deaths globally.^7^

PPH is commonly defined as blood loss within 24 h of ≥500 mL in women who had vaginal birth and ≥1000 mL in women who underwent cesarean section. The impact of labor induction/augmentation on PPH could potentially be exacerbated by anemia during pregnancy. Our systematic review in 2018^8^ identified only one observational study which showed that pregnant women with moderate and severe anemia in India had a higher PPH risk if they had labor induction.^9^ The possible explanations may include uterine muscle fatigue due to excessive uterine stimulation leading to subsequent uterine atony^10^ or a potentially impaired coagulation profile due to iron deficiency among pregnant women with anemia as demonstrated in our previous study.^11^ However, our updated search found a retrospective cohort study from Hong Kong reporting an increased PPH risk among pregnant women with anemia if their labor was not induced or augmented.^12^ Other studies on the association between anemia in pregnancy and PPH have not investigated whether the association varied by status of labor induction/augmentation or their indications.^13^, ^14^, ^15^, ^16^, ^17^, ^18^, ^19^, ^20^, ^21^, ^22^, ^23^, ^24^ Again, a large number of observational studies have examined induction and augmentation of labor in relation to blood loss volume or risk of PPH, but they rarely considered the clinical indications for these interventions and did not explore whether the risk differed by maternal anemia status.^3^, ^25^, ^26^, ^27^, ^28^, ^29^, ^30^, ^31^, ^32^, ^33^, ^34^, ^35^, ^36^, ^37^ Therefore, at present, there is no robust evidence on the safety of induction and augmentation of labor in women with anemia, and how the outcome of labor and birth might be influenced by the severity of anemia and clinical indications for these interventions. As a result, current guidelines have not yet clearly specified whether it is safe to induce and augment labor in pregnant women with anemia.^4^, ^5^, ^6^

This research is particularly relevant for India, given the high prevalence of anemia in pregnancy (50.3%)^38^ and maternal deaths due to PPH (47%).^39^ We previously found high rates of induction and augmentation of labor in our study cohort in India.^1^ One‐third of these labor inductions/augmentations were elective or not clinically indicated^1^ as per national and international guidelines.^4^, ^5^, ^6^ Therefore, the study objectives were to investigate whether induction/augmentation of labor in pregnant women with anemia increases the risk of PPH and whether this risk varied by indications for labor induction/augmentation and by anemia severity during late pregnancy.

## 2 MATERIALS AND METHODS

### 2.1 Study design and population

We conducted a multicentre prospective cohort study through the Maternal and Perinatal Health Research Collaboration, India (MaatHRI), to achieve our study objectives. Further details about MaatHRI can be found in our methodology publication^40^ and secondary analyses^1^, ^41^ from this study available elsewhere. Briefly, MaatHRI is a collaboration of 16 hospitals across six states in India, established in September 2018 to undertake research to improve the health of pregnant women and newborn babies in India. The collaborating hospitals together cover approximately 100 000 births each year.^40^ Pregnant women were recruited from the antenatal clinics during their third trimester of pregnancy (≥28 weeks of gestation) between October 2018 and September 2023 from 13 of the MaatHRI collaborating hospitals (10 government and 3 private) across six Indian states: Assam, Meghalaya, Chhattisgarh, Uttar Pradesh, Himachal Pradesh, and Maharashtra.^1^, ^41^ The selection criteria were women who were at least 18 years of age and in the third trimester of pregnancy and planning a vaginal birth in the participating hospital, and provided written informed consent to participate. Women who planned an elective cesarean section were ineligible for inclusion. All recruited pregnant women were followed up during labor and childbirth and up to 48 h post partum.

### 2.2 Sample size calculation

Findings from our previous study showed that the prevalence of PPH was 2.1% in women who had neither anemia nor induction of labor (the unexposed group).^9^ It was estimated that a sample size of 6750 observations would be required to detect associations with a magnitude of effect sizes of ≥1.5 (a conservative estimate based on our previous study).^9^ This calculation assumes an unexposed to exposed ratio of 2, with 80% power at the 5% level of significance. This was inflated by a factor of 3.13 (the design effect) to account for clustering of observations by hospitals based on an intraclass correlation coefficient of 0.01 and further by 25% to account for loss to follow‐up, confounders, and interaction tests including three subcategories of anemia severity. The final sample size was 8803, rounded up to 9000 pregnant women, comprising 3000 exposed (anemic and labor induced/augmented) and 6000 unexposed (neither anemic nor induced/augmented).

### 2.3 Baseline and follow‐up assessments

At the recruitment visit, information about pregnant women's and their partner's sociodemographic status and lifestyle factors^1^ was collected in person by trained research nurses using structured questionnaires. Nonfasting blood samples were also collected from pregnant women and transported within 72 h to a national reference laboratory for assays using a standardized protocol for all participating hospitals, as comprehensively described in our previous study.^11^ Details about the current pregnancy, obstetric history, and preexisting medical comorbidities were ascertained from the medical records of participants.

At the follow‐up visit, which spanned from labor to childbirth and up to 48 h post partum, measurement of the amount of blood loss was performed using: (i) a calibrated under‐buttocks drape with color‐coded markings placed for up to 2 h in women who had a vaginal birth, and (ii) suction bottles and soaked swabs/sponges for women who underwent emergency cesarean section. Clinical information about PPH diagnosis or blood transfusion within 24 h of childbirth was collected from the hospital records. These methods adhered to the recommendations from the American College of Obstetricians and Gynecologists (ACOG)^42^ and were standardized across all participating hospitals, as fully elaborated in our previous study.^11^ Details about labor and childbirth were also abstracted from medical records using standardized data collection forms.

### 2.4 Postpartum hemorrhage

The outcome of interest was primary PPH diagnosed using the measured amount of blood loss after childbirth (≥500 mL for vaginal births and ≥1000 mL for cesarean sections) and clinician‐diagnosed PPH within 24 h after birth, including history of blood transfusion.^11^

### 2.5 Indications for labor induction/augmentation

The variable for indications for labor induction/augmentation was derived by integrating information from several sources. This included: (i) the status of labor induction and augmentation, which were recorded in two separate binary variables (Yes or No), and (ii) all maternal and fetal clinical conditions in the current pregnancy extracted from medical records. Categories of this variable were: (i) “no labor induction and augmentation,” if neither were reported, (ii) “clinically indicated” if labor induction and/or augmentation was conducted based on any clinical indication as per guidelines, and (iii) “not clinically indicated” (termed as “elective”), if conducted without any reported clinical indication or there was any contraindication, regardless of other clinical conditions, as per guidelines. The clinical indications and contraindications align with guidelines for labor induction from the World Health Organization (WHO),^43^ the National Institute for Health and Care Excellence (NICE),^44^ ACOG,^45^ the Federation of Obstetric and Gynecological Societies of India (FOGSI),^46^ and the National Health Mission (NHM) in India,^4^, ^5^, ^6^, ^47^ as summarized in Table S1. Further details about the classification are provided in a separate study.^1^

We included induction/augmentation as a composite variable because our previous study showed that the clinical indications for induction and augmentation overlapped substantially.^1^

### 2.6 Anemia severity during the third trimester of pregnancy

Hemoglobin concentration in pregnant women was measured from blood samples collected during the third trimester at the point of recruitment. In 121 participants (1.3%), hemoglobin measurements were not available because of inadequate samples or hemolysis, so the values were obtained from the antenatal hemoglobin reports. These hemoglobin concentrations were used to classify the severity of anemia in pregnancy according to the WHO definition for anemia in the third trimester of pregnancy:^48^ (i) no anemia (≥11 g/dL), (ii) mild anemia (10–10.9 g/dL), (iii) moderate anemia (7–9.9 g/dL), and (iv) severe anemia (<7 g/dL). Because of the low numbers of PPH (outcome of interest), “no anemia” and “mild anemia” were combined into a single category.

### 2.7 Statistical analyses

Characteristics of participants were summarized using means with standard deviations for continuous variables, and counts with proportions for categorical variables. We performed *χ* ^2^ tests for categorical variables and *t* tests for continuous variables to compare maternal demographic, healthcare utilization, and socioeconomic characteristics between women with and without PPH, and across anemia severity.

We examined the overall association between anemia severity and PPH, and the association of combined moderate–severe anemia and induction/augmentation of labor with PPH, using multivariable modified Poisson regression analyses adjusting for potential confounders. We then explored whether the associations between clinically indicated and elective labor induction/augmentation and PPH varied by maternal anemia severity in the third trimester. We conducted univariable and multivariable modified Poisson regression analyses, including an interaction term between the “indications for labor induction/augmentation” and “anemia severity” variables. If evidence of statistical interaction (*P* < 0.05) was observed, the association of clinically indicated and elective labor induction/augmentation with PPH was further examined, stratified by women with no/mild, moderate, or severe anemia, using univariable and multivariable modified Poisson regression analyses. Modified Poisson regression is an appropriate method for analysis of data with lower event rates such as PPH in our study.

All multivariable models were adjusted for potential confounders, which were selected a priori as those associated with labor induction or augmentation (as observed in our previous study^1^) and also with PPH (based on other studies, especially in India).^30^, ^33^, ^34^, ^35^, ^49^, ^50^, ^51^ The potential confounders included were age at labor, parity, number of antenatal checkups, duration of iron‐folic acid supplementation, body mass index (BMI) in early pregnancy, and religious background (a measure similar to ethnic background used in other settings). The regression model examining the association between anemia severity and PPH was further adjusted for socioeconomic status and severe complications in the current pregnancy. Findings from regression analyses are presented as risk ratios (RRs) or adjusted RRs (aRRs) with 95% confidence intervals (CIs).

Our primary analyses included women with multiple gestation, as this is one of the indications for labor induction. We repeated our analyses by restricting the study population to singleton pregnancies. We additionally conducted sensitivity analysis by further adjusting the observed findings for mode of childbirth, which included spontaneous vaginal, assisted, and cesarean birth, as a potential mediator. To examine whether the type of methods (mechanical, pharmacological, or combined)^1^ used for induction/augmentation varied by severity of anemia, we conducted a subgroup analysis using multivariable regression including only women who had a labor intervention.

All women who were successfully followed up had complete information about labor induction/augmentation and PPH, as these data fields were mandatory. We assumed the data to be missing at random for variables with missing information, and complete case analysis was performed. *P* values <0.05 were considered statistically significant. All statistical analyses were conducted using Stata version 17.0 (StataCorp LLC).

### 2.8 Ethics statement

This cohort study was performed in accordance with the principles of the Declaration of Helsinki. Ethics approvals were obtained from the institutional review boards of each coordinating Indian institution, the Government of India's Health Ministry's Screening Committee, the Indian Council of Medical Research, New Delhi, and the Oxford Tropical Research Ethics Committee (OxTREC), University of Oxford, UK.

## 3 RESULTS

The cohort study recruited 9420 pregnant women, of whom 128 (1.4%) were excluded, leading to a final sample of 9292 (98.6%) (Figure S1). Reasons for exclusion were loss to follow‐up (*n* = 89, 0.94%), death before childbirth (*n* = 1, 0.01%), opting for elective cesarean section after recruitment (*n* = 25, 0.27%), and/or missing hemoglobin data (*n* = 13, 0.14%). Among the included participants, about half had no labor induction and augmentation (*n* = 4617, 49.7%). In two‐thirds of women who had labor induction/augmentation, the intervention was clinically indicated (*n* = 3083, 66.0%), but in one‐third it was elective or not clinically indicated as per guidelines (*n* = 1592, 34.0%). The elective group mainly comprised women with clinical conditions that were not considered indications according to guidelines. The most common reasons for induction included term pregnancy, anemia, and the desire to accelerate labor and contractions (*n* = 1374, 86.3%). This was followed by those with no recorded clinical condition (*n* = 183, 11.5%) and those with a contraindication (*n* = 35, 2.2%). A substantial number of pregnant women had anemia in their third trimester, including mild (*n* = 1028, 11.1%), moderate (*n* = 5864, 63.1%), and severe (*n* = 1199, 12.9%) anemia, whereas only a small proportion had no anemia (*n* = 1201, 12.9%). Almost all women were married (*n* = 9174, 98.7%) and had a singleton pregnancy (*n* = 9159, 98.6%) who had either: (i) spontaneous vaginal birth (*n* = 3353, 36.6%), (ii) assisted vaginal birth (*n* = 3998, 43.7%) mainly using episiotomy (*n* = 3817, 95.5%), followed by forceps (*n* = 101, 2.5%) and vacuum (*n* = 72, 1.8%), or (iii) emergency cesarean section (*n* = 1808, 19.7%). Among women with multiple gestation (*n* = 133, 1.4%; all twins), more than a quarter had spontaneous vaginal birth for both infants (*n* = 39, 29.3%), or vaginal birth with episiotomy (*n* = 48, 36.1%) or emergency cesarean section (*n* = 46, 34.6%) for at least one of the infants. Almost all women in the cohort had routine active management of third‐stage labor (*n* = 9269, 99.8%) using oxytocin. Approximately 1.2% of the women had a primary PPH (*n* = 113), which were largely atonic PPH (*n* = 80, 70.8%), followed by PPH due to placental problems (*n* = 20, 17.7%); in another 13 women with PPH (11.5%), the reasons were not stated. Only a small proportion of participants had missing data; information about anemia severity and BMI in early pregnancy were missing for 0.1% (*n* = 13) and 4.0% (*n* = 376) of the study cohort, respectively.

Cohort characteristics are presented in Table 1. A higher proportion of women who had a PPH were of Christian religion and had previous pregnancy problems, preexisting medical problems, shorter duration of iron‐folic acid supplementation, multiple gestation in current pregnancy, moderate–severe anemia during the current pregnancy, severe pregnancy complications including gestational hypertension, pre‐eclampsia, eclampsia, antepartum hemorrhage, gestational diabetes and sepsis, preterm birth and emergency cesarean section, or assisted birth using forceps or vacuum, compared with women who did not have a PPH.

**TABLE 1:** Comparisons of characteristics across PPH status in a prospective study in India.

Population characteristics	PPH		
	No (*n* = 9179)	Yes (*n* = 113)	*P* value
Age at labor, mean (SD) (years)	24.75 (4.44)	25.33 (4.89)	0.17
Parity, *n* (%)			0.14
0	6162 (67.1)	69 (61.1)	
1	1939 (21.1)	23 (20.4)	
2–4	1008 (11.0)	19 (16.8)	
≥5	70 (0.8)	2 (1.8)	
Religion, *n* (%)			0.004
Hindu	6765 (73.7)	85 (75.2)	
Muslim	1767 (19.3)	17 (15.0)	
Christian	369 (4.0)	11 (9.7)	
Sikh/others	278 (3.0)	0 (0.0)	
Residence, *n* (%)			0.49
Rural	7849 (85.5)	94 (83.2)	
Suburb/urban	1330 (14.5)	19 (16.8)	
BPL households, *n* (%)			0.48
BPL certificate/self‐certified	4589 (50.0)	62 (54.9)	
Not BPL	3085 (33.6)	32 (28.3)	
Unknown	1505 (16.4)	19 (16.8)	
Woman's education, *n* (%)			0.073
Illiterate	907 (9.9)	9 (8.0)	
Fifth class or below	1908 (20.8)	13 (11.5)	
Sixth to 12th class	4824 (52.6)	73 (64.6)	
Twelfth class or higher	1507 (16.4)	18 (15.9)	
Unknown	33 (0.4)	0 (0.0)	
Husband's occupation, *n* (%)			0.38
Unemployed	582 (6.3)	6 (5.3)	
Partly skilled and unskilled	2749 (29.9)	31 (27.4)	
Skilled manual and nonmanual	5395 (58.8)	66 (58.4)	
Professional, managerial and technical	445 (4.8)	10 (8.8)	
Missing	8 (0.1)	0 (0.0)	
Previous pregnancy problems, *n* (%)			<0.001
No	8704 (94.8)	97 (85.8)	
Yes	342 (3.7)	12 (10.6)	
Unknown	133 (1.4)	4 (3.5)	
Preexisting medical problems, *n* (%)			0.004
No	8969 (97.7)	105 (92.9)	
Yes	209 (2.3)	8 (7.1)	
Missing	1 (0.0)	0 (0.0)	
Adverse lifestyles, *n* (%)			0.059
Never	5963 (65.0)	67 (59.3)	
Past	346 (3.8)	1 (0.9)	
Current	2870 (31.3)	45 (39.8)	
No. of antenatal checkups, *n* (%)			0.85
0–2	1990 (21.7)	28 (24.8)	
3	3244 (35.3)	37 (32.7)	
4	2143 (23.3)	27 (23.9)	
≥5	1802 (19.6)	21 (18.6)	
Duration of iron‐folic acid supplementation, *n* (%) (days)			0.010
None	364 (4.0)	8 (7.1)	
<100	3495 (38.1)	55 (48.7)	
100–179	3998 (43.6)	33 (29.2)	
≥180	1322 (14.4)	17 (15.0)	
Multiple gestation, *n* (%)			0.006
No	9052 (98.6)	107 (94.7)	
Yes	127 (1.4)	6 (5.3)	
BMI in early pregnancy, mean (SD) (kg/m^2^)	20.28 (2.98)	20.67 (3.49)	0.21
Gestational weight gain, mean (SD) (kg/week)	0.30 (0.45)	0.30 (0.55)	0.96
Anemia severity, *n* (%)			<0.001
No (≥11 g/dL)	1192 (13.0)	9 (8.0)	
Mild (10–10.9 g/dL)	1026 (11.2)	2 (1.8)	
Moderate (7–9.9 g/dL)	5789 (63.1)	75 (66.4)	
Severe (<7 g/dL)	1172 (12.8)	27 (23.9)	
Severe complications in current pregnancy, *n* (%)^a^			<0.001
No	8324 (90.7)	79 (69.9)	
Yes	855 (9.3)	34 (30.1)	
Indications for labor induction/augmentation, *n* (%)			0.22
No induction and augmentation	4569 (49.8)	48 (42.5)	
Clinically indicated labor induction/augmentation	3043 (33.2)	40 (35.4)	
Elective labor induction/augmentation	1567 (17.1)	25 (22.1)	
Gestational age at birth, mean (SD) (weeks)	38.64 (2.05)	38.01 (2.61)	0.001
Categories of gestational age at birth, *n* (%)			0.005
Preterm (<37 weeks)	1424 (15.5)	30 (26.5)	
Term (37–40 weeks)	7483 (81.5)	79 (69.9)	
Post‐term (41–44)	272 (3.0)	4 (3.5)	
Active management of third stage of labor, *n* (%)			0.031
No	21 (0.2)	2 (1.8)	
Yes	9158 (99.8)	111 (98.2)	
Mode of childbirth, *n* (%)			<0.001
Spontaneous vaginal birth	3374 (36.8)	18 (15.9)	
Assisted birth	4007 (43.7)	39 (34.5)	
Emergency cesarean section	1798 (19.6)	56 (49.6)	
Assisted birth methods, *n* (%)^b^			0.002
Episiotomy	3833 (95.7)	32 (82.1)	
Forceps	98 (2.5)	3 (7.7)	
Vacuum	68 (1.7)	4 (10.3)	
Unknown	8 (0.2)	0	

Abbreviations: BMI, body mass index; BPL, below the poverty line; PPH, postpartum hemorrhage; SD, standard deviation.

^a^ Including gestational hypertension, pre‐eclampsia, eclampsia, antepartum hemorrhage, gestational diabetes, and sepsis.

^b^ Denominator includes only women with assisted births.

Cohort characteristics by anemia severity are shown in Table S2. Due to the large sample size, many of the characteristics appeared to be statistically significantly different across the categories, although many showed modest differences, such as age at labor, parity, religion, previous pregnancy problems, preexisting medical problems, adverse lifestyles, BMI in early pregnancy, gestational weight gain, severe pregnancy complications, and assisted birth. However, a higher proportion of women who had severe anemia appeared to be from lower socioeconomic status (below poverty line–certified, illiterate, husband in partly skilled or unskilled occupations). They had a lower number of antenatal checkups and shorter duration of iron‐folic acid supplementation. A higher proportion of women with severe anemia underwent clinically indicated labor induction/augmentation, experienced spontaneous vaginal birth, and had preterm birth.

### 3.1 Associations of anemia severity and labor induction/augmentation with PPH

Similar to our previous study,^9^ women with moderate (aRR, 2.12 [95% CI, 1.12–4.01], *P* = 0.020) and severe (aRR, 3.66 [95% CI, 1.80–7.45], *P* < 0.001) anemia had a higher risk of PPH after adjusting for potential confounders, compared with women with no/mild anemia. In addition, women with moderate–severe anemia had a much higher risk of PPH if they had labor induction/augmentation (aRR, 5.32 [95% CI, 1.65–17.13], *P* = 0.005). There was no evidence of a significant association of clinically indicated (RR, 1.25 [95% CI, 0.82–1.89], *P* = 0.298) and elective (RR, 1.51 [95% CI, 0.93–2.44], *P* = 0.092) labor induction/augmentation with PPH. The findings were similar with the adjustment for confounders (clinically indicated: aRR, 1.31 [95% CI, 0.77–2.22], *P* = 0.326; elective: aRR, 1.53 [95% CI, 0.90–2.62], *P* = 0.118). The results are summarized in Table 2.

**TABLE 2:** PPH associated with anemia severity in the third trimester of pregnancy and indications for labor induction/augmentation in a prospective study in India.

	Total	PPH, *n* (%)	Crude model (*n* = 9292)		Adjusted model (*n* = 8916)	
			RR (95% CI)	*P* value	Adjusted RR (95% CI)	*P* value
Anemia severity						
No/mild (≥10 g/dL)	2229	11 (0.5)	1.00		1.00	
Moderate (7–9.9 g/dL)	5864	75 (1.3)	2.59 (1.38–4.87)	0.003	2.12 (1.12–4.01)^a^	0.020
Severe (<7 g/dL)	1199	27 (2.3)	4.56 (2.27–9.17)	<0.001	3.66 (1.80–7.45)^a^	<0.001
Moderate to severe anemia and/or induction/augmentation of labor						
None	1376	3 (0.2)	1.00		1.00	
Either	4094	53 (1.3)	5.94 (1.86–18.97)	0.003	4.32 (1.34–13.87)^b^	0.014
Both	3822	57 (1.5)	6.84 (2.15–21.81)	0.001	5.32 (1.65–17.13)^b^	0.005
Indications for labor induction/augmentation						
No induction and augmentation	4617	48 (1.0)	1.00		1.00	
Clinically indicated labor induction/augmentation	3083	40 (1.3)	1.25 (0.82–1.89)	0.298	1.31 (0.77–2.22)^b^	0.326
Elective labor induction/augmentation	1592	25 (1.6)	1.51 (0.93–2.44)	0.092	1.53 (0.90–2.62)^b^	0.118

Abbreviations: CI, confidence interval; PPH, postpartum hemorrhage; RR, relative risk.

^a^ Adjusted for age at labor, parity, number of antenatal checkups, duration of iron‐folic acid supplementation, body mass index in early pregnancy, religion, households below the poverty line, woman's education, husband's occupation, and severe complications in current pregnancy including gestational hypertension, pre‐eclampsia, eclampsia, antepartum hemorrhage, gestational diabetes, and sepsis.

^b^ Adjusted for age at labor, parity, number of antenatal checkups, duration of iron‐folic acid supplementation, body mass index in early pregnancy, and religion.

### 3.2 Association between indications for labor induction/augmentation and PPH stratified by anemia severity

Associations between the indications for labor induction/augmentation and PPH were found to vary by anemia severity during the third trimester before (crude *P* value for interaction = 0.001) and after (adjusted *P* value for interaction = 0.003) adjustment for confounders.

Table 3 shows the association of clinically indicated and elective labor induction/augmentation with PPH, stratified by anemia severity. Among pregnant women with no/mild anemia, the risk of PPH in both clinically indicated (aRR, 3.81 [95% CI, 0.70–20.81], *P* = 0.123) and elective (aRR, 3.32 [95 CI, 0.74–14.79], *P* = 0.116) labor induction/augmentation groups were not different from the “no induction and augmentation” group. Similarly, among pregnant women with moderate anemia, no evidence of an association between indications of labor induction/augmentation and PPH was observed (clinically indicated: aRR, 1.01 [95% CI, 0.53–1.92], *P* = 0.987; elective: aRR, 0.93 [95% CI, 0.46–1.88], *P* = 0.835). However, among pregnant women with severe anemia, the elective group had more than a three‐fold higher RR of PPH (aRR, 3.44 [95% CI, 1.29–9.18], *P* = 0.014) but there was no evidence of an association in the clinically indicated group (aRR, 1.22 [95% CI, 0.42–3.55], *P* = 0.715). The magnitudes of association remained materially unchanged with the additional adjustment for mode of childbirth (elective: aRR, 3.49 [95% CI, 1.31–9.33], *P* = 0.013; clinically indicated: aRR, 1.17 [95% CI, 0.40–3.39], *P* = 0.774) and after restricting the study population to singleton pregnancies (elective: aRR, 3.97 [95% CI, 1.48–10.60], *P* = 0.006; clinically indicated: aRR, 1.09 [95% CI, 0.34–3.46], *P* = 0.889).

**TABLE 3:** Association of clinically indicated and elective labor induction/augmentation with PPH by anemia severity in the third trimester of pregnancy in a prospective study in India.

	Total	PPH, *n* (%)	Crude model (*n* = 9292)^a^		Adjusted model^b^, ^c^ (*n* = 8916)	
			RR (95% CI)	*P* value	Adjusted RR (95% CI)	*P* value
No/mild anemia (≥10 g/dL)						
No induction and augmentation	1376	3 (0.2)	1.00		1.00	
Clinically indicated labor induction/augmentation	563	5 (0.9)	4.07 (0.98–16.99)	0.054	3.81 (0.70–20.81)	0.123
Elective labor induction/augmentation	290	3 (1.0)	4.75 (0.96–23.40)	0.056	3.32 (0.74–14.79)	0.116
Moderate anemia (7–9.9 g/dL)						
No induction and augmentation	2728	34 (1.3)	1.00		1.00	
Clinically indicated labor induction/augmentation	2004	27 (1.4)	1.08 (0.65–1.79)	0.761	1.01 (0.53–1.92)	0.987
Elective labor induction/augmentation	1132	14 (1.2)	0.99 (0.53–1.84)	0.980	0.93 (0.46–1.88)	0.835
Severe anemia (<7 g/dL)						
No induction and augmentation	513	11 (2.1)	1.00		1.00	
Clinically indicated labor induction/augmentation	516	8 (1.6)	0.72 (0.29–1.78)	0.481	1.22 (0.42–3.55)	0.715
Elective labor induction/augmentation	170	8 (4.7)	2.19 (0.90–5.37)	0.085	3.44 (1.29–9.18)	0.014

Abbreviations: CI, confidence interval; PPH, postpartum hemorrhage; RR, relative risk.

^a^ *P* value for crude interaction between labor induction/augmentation and anemia severity = 0.001.

^b^ *P* value for adjusted interaction between labor induction/augmentation and anemia severity = 0.003.

^c^ Adjusted for age at labor, parity, number of antenatal checkups, duration of iron‐folic acid supplementation, body mass index in early pregnancy, and religion.

Subgroup analysis examining whether the type of methods used for labor interventions varied by anemia status did not show the methods to be different among the anemia severity groups (Table S3).

## 4 DISCUSSION

In this large‐scale multicenter prospective cohort study in India, we found that women with moderate or severe anemia during the third trimester of pregnancy, especially when they had labor induction/augmentation, had a higher risk of PPH. Women who had anemia and underwent induction/augmentation of labor had over five times the risk of PPH than women without anemia who had a spontaneous labor. Further analysis showed that overall, there was no significant association between PPH and indications for labor induction/augmentation, but pregnant women with severe anemia had more than a three‐fold higher risk of PPH if their labor was induced/augmented without any clinical indication (or electively). Conversely, they had no increased PPH risk if labor induction/augmentation was performed in response to clinical indications. Pregnant women with no/mild or moderate anemia did not have an increased risk of PPH whether or not their labor was induced/augmented as per clinical guidelines. These findings were not influenced by mode of childbirth (including whether assisted birth or emergency cesarean section), multiple pregnancies, or type of methods used for labor interventions.

Our findings contribute to new evidence about the safety of labor induction/augmentation, considering the growing evidence of increased risk of PPH among women with anemia.^3^, ^25^, ^26^, ^27^, ^28^, ^29^, ^30^, ^31^, ^32^, ^33^, ^34^, ^35^, ^36^, ^37^ Our earlier study in India (*n* = 1011)^9^ and a recent study in Hong Kong (*n* = 120 129)^12^ showed conflicting associations of anemia in pregnancy with PPH by status of induction or augmentation of labor. The discrepancy could be attributable to different underlying pregnancy complications in the study populations, indicating the need for induction or augmentation of labor, but there was no information about whether induction/augmentation was clinically indicated or elective. By contrast, this prospective study differentiated clinically indicated and elective labor induction/augmentation based on guidelines^4^, ^5^, ^43^, ^44^, ^45^, ^46^, ^47^ and demonstrated that labor intervention without a clear clinical indication in pregnant women with severe anemia could increase the risk of PPH. We were also able to replicate our previous findings of higher PPH risk in women with moderate and severe anemia. In addition, we found a significant interaction between maternal anemia and labor induction/augmentation in relation to PPH.^9^

This study sets the foundation for further research to advance current national and international guidelines^43^, ^44^, ^45^, ^46^, ^47^ on labor induction/augmentation for pregnant women with anemia. Globally, the prevalence of anemia in pregnancy is notably high, affecting 37% of pregnancies, with over a quarter of these cases diagnosed as moderate (26.5%) or severe (2.3%) anemia.^52^ Despite this, current guidelines^4^, ^5^, ^43^, ^44^, ^45^, ^46^, ^47^ do not specify whether labor induction/augmentation is safe for pregnant women with anemia, nor do they outline the conditions under which these interventions may be deemed inappropriate. Our findings suggest that induction/augmentation of labor may be generally safe for pregnant women with anemia. However, extra caution may be warranted when considering labor induction/augmentation for pregnant women with severe anemia in the absence of a clear clinical indication. These findings warrant the replication of similar studies in other settings with more comprehensive outcome data, as well as further investigation into the potential biological mechanisms underlying the higher risk of PPH in women undergoing elective labor induction/augmentation.

There could be several potential explanations for our observations. In the presence of clinical indications, the benefit of labor induction/augmentation might outweigh the risk of PPH associated with moderate to severe anemia in pregnancy.^9^ Conversely, if women with severe anemia are unnecessarily subjected to labor interventions that increase uterine contractility, it could result in early uterine muscle fatigue from insufficient oxygen supply due to very low hemoglobin levels, leading to uterine atony and PPH.^9^ In addition, as shown in our previous study, anemia in pregnancy may contribute to alterations in the coagulation profile, including higher D‐dimer and lower fibrinogen levels and a higher international normalized ratio, thereby raising the risk of excessive bleeding.^11^ However, the exact mechanisms by which indications for labor induction/augmentation and anemia in pregnancy interact and contribute to PPH risk are unclear. Alternatively, there could be differential third‐stage labor management among women with severe anemia, but nearly all women in the study cohort received active management of the third stage of labor, and the type of method used for labor interventions did not vary by anemia status. Furthermore, the observed findings did not change after accounting for mode of childbirth or by restricting the study population to singleton pregnancies.

The main strength of this study lies in its prospective data collection approach in a large sample of women in India. This includes the measurement of hemoglobin concentration during the third trimester of pregnancy, standardized assessment of PPH from blood loss volume and clinical diagnosis to minimize uncertainty in variation between individuals, and the standardized extraction of all maternal and fetal clinical conditions from medical records. These minimize potential selection bias, reporting bias and reverse causality. However, we cannot completely exclude the possibility of misclassifications of the indications for labor induction/augmentation because of incomplete recording of maternal or fetal conditions in medical records. We observed a small number of women with PPH (*n* = 113, 1.2%) due to the low prevalence of this complication in our study population (1.1% to 1.6%).^11^, ^40^, ^53^ Although we had adequate statistical power for the overall analysis, the null associations observed in the stratified analyses, especially among women with no/mild anemia, could be attributable to the small sample sizes in those subgroups, which result in limited power. Measurement errors may exist in the measures of hemoglobin concentrations, despite employing standardized laboratory procedures. These would, however, only lead to a nonsystematic bias, which is unlikely to affect our findings. Furthermore, our findings may be subject to residual confounding caused by unknown factors, although our analyses were adjusted for several potential confounders identified a priori from previous studies and the included study cohort did not have a reported history of coagulation disorders, disseminated intravascular coagulopathy, or other disorders that may increase the risk of PPH.^11^ We cannot rule out the possibility of chance findings in the interaction and stratification analyses, but the current analyses are informed by our previous studies^9^, ^11^ and also replicate our previous findings.^9^ Finally, while our analyses included 13 hospitals across six states in India, caution is warranted when generalizing our findings to other settings that may follow different guidelines for labor induction/augmentation. Nevertheless, we have considered multiple guidelines^4^, ^5^, ^43^, ^44^, ^45^, ^46^, ^47^ to encompass a broader range of potential clinical indications and contraindications for labor induction to minimize misclassification. We were also able to replicate the higher risk of PPH in pregnant women with moderate and severe anemia, suggesting that our study cohort is comparable to the populations in our previous study^9^ and possibly other studies that demonstrated similar risk.^54^, ^55^, ^56^

In conclusion, while induction/augmentation of labor may be generally safe for women with anemia, it may result in a higher risk of PPH in women with severe anemia if performed electively. These findings highlight the urgent need for further research to assess whether severe anemia in pregnancy could be a relative contraindication for labor induction/augmentation in the absence of a clinical indication. Therefore, our study suggests the need to ascertain clinical indications for these interventions in this high‐risk group. If a need for labor induction/augmentation is ascertained for women with severe anemia despite a lack of clear clinical indication, the third stage of labor should be actively managed and the clinical team should be prepared to identify bleeding early and manage it appropriately. Importantly, to reduce the risk of PPH in women with moderate–severe anemia, which cannot be completely explained by labor interventions, it is important to correct hemoglobin levels in pregnant women before they go into labor. Considering the global widespread practice of labor induction/augmentation,^1^, ^2^, ^3^, ^57^, ^58^ as well as high worldwide rates of anemia in pregnancy^52^ and maternal deaths due to PPH,^58^ it is important to undertake more research to update local and global guidelines for labor induction/augmentation.

## AUTHOR CONTRIBUTIONS

**Tuck Seng Cheng:** Conceptualization; data curation; formal analysis; investigation; data analysis; interpretation of data; writing‐original draft; writing‐review and editing. **Farzana Zahir:** Conceptualization; data curation; project administration; interpretation of data; writing‐review and editing. **Carolin V. Solomi:** Conceptualization; data curation; project administration; interpretation of data; writing‐review and editing. **Ashok Verma:** Conceptualization; data curation; project administration; interpretation of data; writing‐review and editing. **Sereesha Rao:** Conceptualization; data curation; project administration; interpretation of data; writing‐review and editing. **Saswati Sanyal Choudhury:** Conceptualization; data curation; project administration; interpretation of data; interpretation of data; writing‐review and editing. **Gitanjali Deka:** Conceptualization; data curation; project administration; interpretation of data; writing‐review and editing. **Pranabika Mahanta:** Conceptualization; data curation; project administration; interpretation of data; writing‐review and editing. **Swapna Kakoty:** Conceptualization; data curation; project administration; interpretation of data; writing‐review and editing. **Robin Medhi:** Conceptualization; data curation; project administration; interpretation of data; writing‐review and editing. **Shakuntala Chhabra:** Conceptualization; data curation; project administration; interpretation of data; writing‐review and editing. **Anjali Rani:** Conceptualization; data curation; project administration; interpretation of data; writing‐review and editing. **Amrit Bora:** Conceptualization; data curation; project administration; interpretation of data; writing‐review and editing. **Indrani Roy:** Conceptualization; data curation; project administration; interpretation of data; writing‐review and editing. **Bina Minz:** Conceptualization; data curation; project administration; interpretation of data; writing‐review and editing. **Omesh Kumar Bharti:** Interpretation of data; writing‐review and editing. **Rupanjali Deka:** Data curation; project administration; writing‐review and editing. **Charles Opondo:** Interpretation of data; writing‐review and editing. **David Churchill:** Interpretation of data; writing‐review and editing. **Marian Knight:** Methodology; writing‐review and editing. **Jennifer J. Kurinczuk:** Methodology; writing‐review and editing. **Manisha Nair:** Conceptualization; data curation; supervision; methodology; investigation; interpretation of data; writing‐review and editing.

## FUNDING INFORMATION

The MaatHRI platform and this study was funded by a Medical Research Council (grant number MR/P022030/1) and a Transition Support Award (grant number MR/W029294/1) for MN.

## CONFLICT OF INTEREST STATEMENT

The authors have no conflicts of interest.

## Supporting information


Data S1.

## Data Availability

Research data are available upon reasonable request by contacting the corresponding author.
