# Supplementary material for: Does induction or augmentation of labor increase the risk of postpartum hemorrhage in pregnant women with anemia? A multicenter prospective cohort study in India
Source: Int J Gynaecol Obstet. 2024 Nov 8;169(1):299–309. doi: 10.1002/ijgo.16008 (PMC11911977; doi:10.1002/ijgo.16008)
Supplement: Supplementary file 1 — Data S1. [file IJGO-169-299-s001.docx]

| **Table S1. Maternal and fetal indications and contraindications for induction of labour according to guidelines^a^** | |
| --- | --- |
| **Indications** | **Contraindications** |
| - Pre-existing or current pregnancy health problems - Pre-existing diabetes mellitus or gestational diabetes mellitus - Hypertension disorders in pregnancy including pre-existing hypertension, pregnancy-induced hypertension, preeclampsia and eclampsia - Antepartum haemorrhage - Premature rupture of membrane - Chorioamnionitis - Oligohydramnios - Obstetric cholestasis - Isoimmunisation - Renal disease - Cardiac problem - Current fetal health problems - Structural defects - Intrauterine demise - Small for gestational age (based on birthweight <2500g) - Post-term pregnancy (≥41 weeks of gestation calculated from last menstrual period) - Multiple gestation | - Specific placental problems such as a major placenta praevia - ≥2 previous caesarean sections - Fetal malpresentation including breech presentation |
| ^a^the World Health Organization,^42^ the National Institute for Health and Care Excellence,^43^ the American College of Obstetricians and Gynecologists,^44^ the Federation of Obstetric and Gynecological Societies of India,^45^ and the National Health Mission (NHM) in India^4,5,46^ | |

**Figure S1. Flow chart for the inclusion of the study population**

9,420 recruited

9,305

- Loss to follow-up (n=89)
- Death before childbirth (n=1)
- Opting for elective caesarean sections after recruitment (n=25)

Missing haemoglobin concentrations (n=13)

9,292

| **Table S2. Comparisons of characteristics across anaemia severity status in a prospective study in India** | | | | |  |
| --- | --- | --- | --- | --- | --- |
| **Population Characteristics** | **Anaemia severity** | | | | |
|  | **No/mild (n=2229)** | **Moderate (n=5864)** | **Severe (n=1199)** | **P Value** | |
| Age at labour (years) (mean, SD) | 24.55 (4.10) | 24.76 (4.54) | 25.15 (4.60) | <0.001 | |
| Parity (n, %) |  |  |  | <0.001 | |
| 0 | 1,589 (71.3) | 3,854 (65.7) | 788 (65.7) |  | |
| 1 | 460 (20.6) | 1,271 (21.7) | 231 (19.3) |  | |
| 2-4 | 176 (7.9) | 679 (11.6) | 172 (14.3) |  | |
| ≥5 | 4 (0.2) | 60 (1.0) | 8 (0.7) |  | |
| Religion (n, %) |  |  |  | <0.001 | |
| Hindu | 1,668 (74.8) | 4,300 (73.3) | 882 (73.6) |  | |
| Muslim | 407 (18.3) | 1,124 (19.2) | 253 (21.1) |  | |
| Christian | 46 (2.1) | 286 (4.9) | 48 (4.0) |  | |
| Sikh/others | 108 (4.8) | 154 (2.6) | 16 (1.3) |  | |
| Residence (n, %) |  |  |  | 0.63 | |
| Rural | 1,893 (84.9) | 5,018 (85.6) | 1,032 (86.1) |  | |
| Suburb/urban | 336 (15.1) | 846 (14.4) | 167 (13.9) |  | |
| Below poverty line (BPL) households (n, %) |  |  |  | <0.001 | |
| BPL certificate/self-certified | 917 (41.1) | 2,934 (50.0) | 800 (66.7) |  | |
| not BPL | 819 (36.7) | 2,003 (34.2) | 295 (24.6) |  | |
| Unknown | 493 (22.1) | 927 (15.8) | 104 (8.7) |  | |
| Woman’s education (n, %) |  |  |  | <0.001 | |
| Illiterate | 212 (9.5) | 526 (9.0) | 178 (14.8) |  | |
| ≤5th class | 433 (19.4) | 1,129 (19.3) | 359 (29.9) |  | |
| 6-12th class | 1,103 (49.5) | 3,261 (55.6) | 533 (44.5) |  | |
| ≥12th class | 474 (21.3) | 927 (15.8) | 124 (10.3) |  | |
| Unknown | 7 (0.3) | 21 (0.4) | 5 (0.4) |  | |
| Husband’s occupation (n, %) |  |  |  | <0.001 | |
| Unemployed | 233 (10.5) | 307 (5.2) | 48 (4.0) |  | |
| Partly skilled and unskilled | 498 (22.3) | 1,760 (30.0) | 522 (43.5) |  | |
| Skilled manual and non-manual | 1,382 (62.0) | 3,489 (59.5) | 590 (49.2) |  | |
| Professional, managerial and technical | 113 (5.1) | 303 (5.2) | 39 (3.3) |  | |
| missing | 3 (0.1) | 5 (0.1) | 0 (0.0) |  | |
| Previous pregnancy problems (n, %) |  |  |  | <0.001 | |
| No | 2,160 (96.9) | 5,528 (94.3) | 1,113 (92.8) |  | |
| Yes | 48 (2.2) | 239 (4.1) | 67 (5.6) |  | |
| Unknown | 21 (0.9) | 97 (1.7) | 19 (1.6) |  | |
| Pre-existing medical problems (n, %) |  |  |  | <0.001 | |
| No | 2,192 (98.3) | 5,732 (97.7) | 1,150 (95.9) |  | |
| Yes | 37 (1.7) | 132 (2.3) | 48 (4.0) |  | |
| missing | 0 (0.0) | 0 (0.0) | 1 (0.1) |  | |
| Adverse lifestyles (n, %) |  |  |  | <0.001 | |
| Never | 1,532 (68.7) | 3,751 (64.0) | 747 (62.3) |  | |
| Past | 97 (4.4) | 184 (3.1) | 66 (5.5) |  | |
| Current | 600 (26.9) | 1,929 (32.9) | 386 (32.2) |  | |
| Number of antenatal check-ups (n, %) |  |  |  | <0.001 | |
| 0-2 | 502 (22.5) | 1,191 (20.3) | 325 (27.1) |  | |
| 3 | 707 (31.7) | 2,076 (35.4) | 498 (41.5) |  | |
| 4 | 545 (24.5) | 1,410 (24.0) | 215 (17.9) |  | |
| ≥5 | 475 (21.3) | 1,187 (20.2) | 161 (13.4) |  | |
| Duration of iron-folic acid supplementation (days) (n, %) |  |  |  | <0.001 | |
| None | 53 (2.4) | 230 (3.9) | 89 (7.4) |  | |
| <100 | 618 (27.7) | 2,247 (38.3) | 685 (57.1) |  | |
| 100-179 | 1,094 (49.1) | 2,612 (44.5) | 325 (27.1) |  | |
| ≥180 | 464 (20.8) | 775 (13.2) | 100 (8.3) |  | |
| Multiple gestation (n, %) |  |  |  | 0.13 | |
| No | 2,207 (99.0) | 5,772 (98.4) | 1,180 (98.4) |  | |
| Yes | 22 (1.0) | 92 (1.6) | 19 (1.6) |  | |
| BMI in early pregnancy (kg/m^2^) (mean, SD) | 20.45 (3.28) | 20.29 (2.93) | 19.95 (2.61) | <0.001 | |
| Gestational weight gain (kg/week) (mean, SD) | 0.29 (0.43) | 0.31 (0.47) | 0.27 (0.37) | 0.035 | |
| Severe complications in current pregnancy^a^ (n, %) |  |  |  | 0.003 | |
| No | 1,980 (88.8) | 5,316 (90.7) | 1,107 (92.3) |  | |
| Yes | 249 (11.2) | 548 (9.3) | 92 (7.7) |  | |
| Indications for labour induction/augmentation (n, %) |  |  |  | <0.001 | |
| No induction and augmentation | 1,376 (61.7) | 2,728 (46.5) | 513 (42.8) |  | |
| Clinically indicated labour induction/augmentation | 563 (25.3) | 2,004 (34.2) | 516 (43.0) |  | |
| Elective labour induction/augmentation | 290 (13.0) | 1,132 (19.3) | 170 (14.2) |  | |
| Gestational age at birth (weeks) (mean, SD) | 38.75 (1.95) | 38.64 (2.03) | 38.42 (2.38) | <0.001 | |
| Categories of gestational age at birth (n, %) |  |  |  | <0.001 | |
| Preterm (<37 weeks) | 325 (14.6) | 886 (15.1) | 243 (20.3) |  | |
| Term (37-40 weeks) | 1,826 (81.9) | 4,817 (82.1) | 919 (76.6) |  | |
| Post-term (41-44) | 78 (3.5) | 161 (2.7) | 37 (3.1) |  | |
| Active management of third stage of labour (n, %) |  |  |  | 0.21 | |
| No | 2 (0.1) | 18 (0.3) | 3 (0.3) |  | |
| Yes | 2,227 (99.9) | 5,846 (99.7) | 1,196 (99.7) |  | |
| Mode of childbirth (n, %) |  |  |  | <0.001 | |
| Spontaneous vaginal birth | 770 (34.5) | 2,140 (36.5) | 482 (40.2) |  | |
| Assisted birth | 987 (44.3) | 2,503 (42.7) | 556 (46.4) |  | |
| Emergency caesarean section | 472 (21.2) | 1,221 (20.8) | 161 (13.4) |  | |
| Assisted birth methods (n, %)* |  |  |  | 0.006 | |
| Episiotomy | 953 (96.6) | 2380 (95.1) | 532 (95.7) |  | |
| Forceps | 28(2.8) | 59 (2.4) | 14 (2.5) |  | |
| Vacuum | 5 (0.5) | 57 (2.3) | 10 (1.8) |  | |
| Unknown | 1 (0.1) | 7 (0.3) | 0 |  | |
| SD, standard deviation; n, number; %, percentage  ^a^including gestational hypertension, pre-eclampsia, eclampsia, antepartum haemorrhage, gestational diabetes mellitus and sepsis  *Denominator includes only women with assisted births | | | | |  |

| **Table S3. Association of anaemia severity in the third trimester of pregnancy with methods for labour induction/augmentation in a prospective study in India** | | |  |  |
| --- | --- | --- | --- | --- |
|  | **Crude model (N=4675)** | | | |
|  | **RR (95% CI)** | **P value** | |  |
| Mechanical only | Base outcome | | |  |
| Pharmacological only |  |  | |  |
| Anaemia severity |  |  | |  |
| No/mild (≥10 g/dL) | 1.00 |  | |  |
| Moderate (7-9.9 g/dL) | 0.93 (0.77, 1.12) | 0.422 | |  |
| Severe (<7 g/dL) | 1.10 (0.86, 1.41) | 0.434 | |  |
| Combination |  |  | |  |
| Anaemia severity |  |  | |  |
| No/mild (≥10 g/dL) | 1.00 |  | |  |
| Moderate (7-9.9 g/dL) | 0.95 (0.76, 1.19) | 0.667 | |  |
| Severe (<7 g/dL) | 0.75 (0.55, 1.03) | 0.073 | |  |
| RR, relative risk; CI, confidence interval | | |  |  |
